# In vitro evaluation of methicillin-resistant and methicillin-sensitive *Staphylococcus aureus* susceptibility to Saudi honeys

**DOI:** 10.1186/s12906-019-2603-8

**Published:** 2019-07-25

**Authors:** Muhammad Barkaat Hussain, Yasser Mahmoud Kamel, Zia Ullah, Asif Ahmad Mohamad Jiman-Fatani, Ansar Shafiq Ahmad

**Affiliations:** 1 Department of Microbiology, Faculty of Medicine, King Abdulaziz University, Rabigh Branch, 21589 Saudi Arabia; 2 Department of Medical Microbiology and Parasitology, Faculty of Medicine, King Abdulaziz University, Rabigh branch, 21589 Saudi Arabia; 3 Department of Surgery, Faculty of Medicine, King Abdulaziz University, Rabigh branch, 21589 Saudi Arabia

**Keywords:** Wounds, Infection, Antibacterial activity, Sumra honey, MRSA

## Abstract

**Background:**

Honey has been increasingly recognized as a potential therapeutic agent for treatment of wound infections. There is an urgent need for assessment and evaluation of the antibacterial properties against wound pathogens of honeys that have not yet been tested.

**Methods:**

Ten Saudi honeys collected from different geographical locations were screened initially for their antibacterial potential against methicillin-resistant *Staphylococcus aureus* (MRSA) and methicillin-sensitive *Staphylococcus aureus* (MSSA) by the agar well diffusion method. Manuka honey (UMF-12) was used for comparison. Of the tested honeys, the honey that exhibited the greatest antibacterial activity in the agar well diffusion assay was further evaluated for its minimum inhibitory concentration (MIC) against ten MRSA clinical isolates and three American Type Culture Collection (ATCC) reference strains by the microbroth dilution method.

**Results:**

Locally produced honeys exhibited variable antibacterial activity against the tested isolates in the agar well diffusion assay. They were unable to exhibit antibacterial activity against MSSA and MRSA at 25% dilutions (w/v) in catalase solution. However, Sumra and Talha honeys showed a zone of inhibition at 50% dilutions (w/v) in catalase solution. This finding means that both honeys possess weak non-peroxide-based antibacterial activity. Moreover, Sumra honey showed a larger inhibition zone at 50 and 25% dilutions (w/v) in distilled water than Manuka honey against both MSSA and MRSA. This result demonstrates that Sumra honey has more hydrogen peroxide-related antibacterial activity or total antibacterial activity than Manuka honey. In addition, MIC results obtained through a microbroth dilution assay showed that Sumra honey inhibited the growth of all MRSA clinical isolates (*n* = 10) and reference strains [MRSA (ATCC 43300) and MSSA (ATCC 29213)] at lower concentrations (12.0% v/v) than those required for Manuka honey-mediated inhibition (14.0% v/v). This result means that Sumra honey has more peroxide or synergistic antibacterial activity than Manuka honey. An equivalent MIC (15.0% v/v) was observed for *E. coli* (ATCC 25922) between Manuka honey and Sumra honey.

**Conclusions:**

Sumra honey may be used as an alternative therapeutic agent for infected wounds and burns, where additional hydrogen peroxide-related antibacterial activity is needed. In the future, the physiochemical characteristics of Sumra honey may be evaluated and standardized.

## Background

Human isolates of methicillin-resistant *Staphylococcus aureus* (MRSA) are some of the bacteria most frequently involved in wound infections [1]. MRSA infection is associated with a prolonged healing duration, a rise in postoperative complications, and increased mortality [2]. According to a recent meta-analysis report extracted from seven articles, the overall MRSA prevalence in Saudi Arabia is 38%, which is quite high in comparison with Gulf Corporation Council (GCC) countries, with Kuwait having the lowest (3.3%) [3, 4]. A significant variation exists in the prevalence of MRSA worldwide, which ranges from 12 to 73%. A cross-sectional study in nine European countries revealed that Hungary had a prevalence rate of 12.1% and Sweden had a prevalence rate of 29.4% [5]. New antimicrobial agents are not being produced as quickly as they are needed [6, 7]. Honey, in this regard, is considered to be a promising agent [8]. Honeys collected from different areas have been demonstrated to have substantial antibacterial effects on infected wounds and burns [9, 10]. In addition to its potent antibacterial effects, honey is also useful in reducing inflammation and promoting wound debridement, angiogenesis, granulation, and epithelialization [11]*.* Its effectiveness has been shown in multiple reports regarding a variety of different wounds, including infected wounds, soft tissue infections, burns and skin ulcers [12–14].

A number of clinical trials have shown that the use of honey as a wound dressing is better than the use of topical or systemic antibiotics, including for diabetic foot ulcers [15–18]. Manuka honey-impregnated dressings are effective in even recalcitrant cases that had been previously treated with conventional modalities, such as systemic antibiotic therapy, negative pressure vacuum-assisted dressings, continuous dressing change with local debridement and maggot treatment [19]. A Cochrane systematic review by Jull et al. (2015) concluded that the use of honey in cases other than partial thickness burns and infected postoperative wounds is not supported by high-quality evidence and therefore does not have a strong basis for decision making [20]. One clinical trial has shown that honey dressings did not enhance venous leg ulcer healing in comparison with conventional treatment; rather, honey treatment was associated with more adverse effects and involved higher cost than conventional therapy [21].

Honey has demonstrated multiple antibacterial properties against different wound pathogens, but only a narrow range of medically graded honeys are available for wound management [22–25]. Moreover, certified honeys (approved and registered by health regulatory authorities) are not easily accessible and are generally costly. Therefore, it is important to evaluate new honeys with high levels of antibacterial activity that are locally produced and affordable. Previously, we reported the susceptibility of multidrug-resistant *Salmonella typhi* to honey and conducted a clinical trial of impregnated honey dressings for the treatment of diabetic foot ulcers [17, 26]. Honey is widely consumed in Saudi Arabia as a preventive and curative agent for several human illnesses in addition to its popular usage as food [27]. A number of studies have been performed in Saudi Arabia regarding the antibacterial properties of indigenous honey against different bacterial isolates [28–32]. However, in most of the previous studies, the precise geographical locations of tested honeys were not mentioned, and their antibacterial activity was not compared with medically graded honey [28, 29]. Therefore, keeping in view of these limitations, we tested Saudi honeys with known geographical origins and compared their antibacterial activity with medically graded Manuka honey.

## Methods

### Honey samples

Ten honey samples produced by *Apis mellifera jemenitica* were collected from different geographical locations of Saudi Arabia (Table 1). *Apis mellifera jemenitica* is the native bee in the Arabian Peninsula and is tolerant to local dry and hot weather conditions. The honeys were kept in brown bottles at room temperature, and their sterility was determined on blood agar medium. A loopful (10 μl) quantity of honey from each honey sample was inoculated on blood agar plates. The streaked plates were incubated aerobically at 37 °C and examined for any growth after 24 h. Medically graded Manuka honey (Molan Gold Standard 12 plus, methylglyoxal-400 mg per kg, Watson and Son-New Zealand) purchased from Al-Nahdi Pharmacy, Jeddah, was used for comparison. Artificial honey was also used to evaluate the role of sugar in the antibacterial potential of honey. The artificial honey was prepared by a previously described method [33].

**Table 1:** Geographical location, floral source and harvesting season of Saudi honeys

Code no.	Name of honey	Floral source	Geographical location and coordinates	Harvesting season
H01	Sidr	*Ziziphus spina-christi*	Al-Baha, Baraha Magamaa 20° 0′ 32.94″ N 41° 27′ 53.82″ E	October, 2016
H02	Talha	*Acacia* sp.	Al-Baha, Beta Valley 20.0129° N 41.4677° E	April, 2016
H03	Sumra	*Acacia tortilis*	Al-Baha, Beta Valley 20.0129° N 41.4677° E	April, 2016
H04	Sidr	*Ziziphus spina-christi*	Al-Baha, Baljurashi 19° 50′ 33.828″ N 41° 33′ 43.848″ E	October, 2015
H05	Talha	*Acacia* sp.	Al-Baha, Baljurashi 19° 50′ 33.828″ N 41° 33′ 43.848″ E	April, 2015
H06	Sumra	*Acacia tortilis*	Al-Mukhwah, 19° 47′ 21.48″ N 41° 26′ 22.164″ E	April, 2015
H07	Zahoor	Mixed flora	Ali-al-Saalam, Al-Hasa-Qweiba 30° 57′ 25.74″ N 35° 45′ 46.26″ E	March, 2015
H08	Zahoor	Mixed flora	Ali-al-Khamis, Al-Hasa-Qweiba 30° 41′ 32.676″ N 35° 51′ 51.732″ E	March, 2015
H09	Zahoor	Mixed flora	Muhammad, Al-Hasa-Qweiba 30° 41′ 32.676″ N 35° 51′ 51.732″ E	March, 2015
H11	Sidr	*Ziziphus spina-christi*	Al-Baha, Wadi Beedah and Mahshooqa 20° 0′ 32.94″ N 41° 27′ 53.82″ E	October, 2015

### Bacterial strains

Thirteen bacterial isolates comprising ten clinical isolates of MRSA and three standard strains (ATCC) were used (Table 4). The clinical isolates and reference strains were obtained from King Abdulaziz University Hospital, Clinical and Molecular Microbiology Laboratory, Jeddah, Saudi Arabia.

### Agar well diffusion assay

The agar well diffusion method was used for an initial screening of honey samples, as performed by Allen et al*,* with slight adjustments [34]. The assay is the most frequently used method for assessing the antibacterial potency of honey because of its ease, low cost and rapidity [35]. The details of this procedure were kindly provided by Kerry Allen (personal communication), Honey Research Unit, Waikato University, New Zealand.

Mueller-Hinton (MH) agar (Oxoid Ltd., UK) was used instead of nutrient agar because MH agar is the preferred medium for antibacterial testing according to the CLSI standard [36]. Second, we used vancomycin (30 μg) as a positive control instead of 6% phenol. A catalase solution and sterile distilled water were used as negative controls.

On sheep blood agar plates, MRSA and MSSA were sub-cultured. From the overnight culture, a 0.5 McFarland turbidity suspension (540 nm) was prepared in tryptic soy broth using a colorimeter. A volume of 100 μl of the culture (0.5 absorbance) was used to inoculate 150 ml of MH medium for each culture. The cultures were kept at 45 °C in a water bath for 25 min before inoculating with 100 μl of each culture. Thoroughly mixed agar was poured into 90 mm diameter Petri dishes (20 ml in each) and stored at 4 °C overnight. Five wells were made in the agar plate according to a standard template with a sterile 9 mm cork borer and were numbered at random.

From each honey sample, 50% (w/v) honey dilutions were prepared in sterile purified water and catalase (Sigma C1345-10G, bovine liver, 5,000 units/mg). To prepare the catalase solution, 20 mg of catalase was added to 10 ml of sterile distilled water. Secondary honey solutions comprising 25% (w/v) honey in sterile purified water and catalase solution were prepared from the primary solution. One hundred twenty microlitre solutions from each dilution were added to each allotted well, and the agar plates were placed in an incubator for 16 h at 35 °C. The inhibition zone was measured in mm. Each experiment was performed in triplicate on the same day using three identical wells.

### Microbroth dilution assay

MICs of Sumra, Manuka and artificial honey against all the clinical isolates of MRSA (*n* = 10) and three ATCC reference strains (Table 4) were determined by a microbroth dilution assay in sterile 96-well microtitre plates (Thermo Fisher Scientific, UK). A 50% (v/v) stock solution of each honey sample was prepared by weighing 13.7 g of the honey and bringing the volume up to 20 ml using Mueller Hinton (MH) broth. Since honey is very viscous and it is difficult to pipette, it was weighed out. The density of honey was as assumed to be 1.37 g/ml [37]. Further, twenty incremental dilutions (from 1 to 20% v/v) were prepared in 96-well microtitre trays by adding a calculated volume of honey from the 50% (v/v) stock solution and a calculated volume of bacterial suspension (5 × 10^5^ CFU/ml) to obtain a final volume of 200 μl in each allotted well. In previous studies, two-fold dilutions of honey were prepared in a microbroth dilution assay to determine the MICs of honey [38, 39]. However, in this study, 1% incremental dilutions were used to obtain more precise inhibitory concentrations of tested honey samples. However, 5% incremental dilutions of artificial honey were prepared, ranging from 5 to 40%.

Five isolated colonies were picked from overnight blood agar culture and inoculated into nutrient broth to obtain a turbidity matched with 0.5 McFarland (1 × 10^8^ CFU/ml). Further dilutions of bacterial suspensions were performed in MH broth to obtain a final dilution of inoculums of 5× 10^5^ CFU/ml. Positive control wells contained MH broth with the bacterial suspension, and negative wells contained MH broth only. The microtitre plates were incubated aerobically at 37 °C for 16 h in a stationary incubator and observed visually for the absence or presence of growth by comparison to the positive and negative controls. The minimum inhibitory concentrations were calculated as the lowest concentration of honey that prevented visible bacterial growth after overnight incubation. All experiments were performed in triplicate on the same day using three identical wells*.*

### Statistical analysis

The data were analysed by IBM Statistical Package for Social Sciences software (SPSS 23.0). The arithmetic mean of the inhibition zone of each honey sample and the MICs of the tested honeys were calculated. The differences among the mean MICs of the tested honeys were calculated by applying the Kruskal-Wallis test. However, the Bonferroni post hoc test was applied for pair-wise comparisons between different honeys. The results were considered significant at *p* < 0.05.

## Results

All locally produced honeys showed antibacterial activity against the MSSA and MRSA clinical isolates and ATCC reference strains, MRSA (43300), MSSA (43300) and *Escherichia coli* (25922), in an agar well diffusion assay (Tables 2 and 3). However, there was much variation in the potency of the antibacterial activity of the tested honeys. The variation existed not only between different floral honeys but also between honeys with the same floral origins (Tables 2 and 3). For instance, Sumra honey (H03) collected from Al-Baha, Beta Valley, produced an inhibition zone of 18.3 ± 0.3 mm, and Sumra honey (H06) collected from Mukhwah exhibited an inhibition zone of 15.2 ± 0.4 mm against MSSA at a 50% dilution in sterile distilled water. Variations also existed in other dilutions. Similarly, there was variation in the level of antibacterial activity of Sidr, Talha and Zahoor honeys against tested pathogens (Tables 2 and 3). The reason for variation in the potency of antibacterial activity of honey sharing a floral origin could be due to climatic conditions, soil composition and geographical areas of honey collection [26]. Variation in the level of antibacterial activity of honeys between different floral honey sources and within the same flora source has been reported in other studies as well [40, 41].

**Table 2:** Inhibition zone (mm) of honey samples at 50 and 25% (w/v) dilutions in sterile purified water and 50 and 25% (v/v) dilutions in catalase solution by agar well diffusion assay against MSSA

Code no.	Honey samples	Zone of inhibition (mm)			
		50% in water	50% in catalase	25% in water	25% in catalase
Standard	Manuka	16.8 ± 0.1	15.4 ± 0.4	12.5 ± 0.2	11.1 ± 0.1
H01	Sidr	16.7 ± 0.2	NZD^a^	11.3 ± 0.2	NZD
H02	Talha	11.3 ± 0.0	NZD	10.1 ± 0.0	NZD
H03	Sumra	18.3 ± 0.3	11.4 ± 0.4	13.0 ± 0.0	NZD
H04	Sidr	16.9 ± 0.1	NZD	10.9 ± 0.0	NZD
H05	Talha	15.5 ± 0.4	11.8 ± 0.3	11.2 ± 0.0	NZD
H06	Sumra	15.2 ± 0.4	NZD	10.0 ± 0.0	NZD
H07	Zahoor	11.5 ± 0.1	NZD	NZD	NZD
H08	Zahoor	18.0 ± 0.1	NZD	12.0 ± 0.0	NZD
H09	Zahoor	13.9 ± 0.2	NZD	10.2 ± 0.1	NZD
H11	Sidr	14.1 ± 0.2	NZD	10.0 ± 0.1	NZD

^a^NZD; no zone detected

**Table 3:** Inhibition zone (mm) of honey samples at 50 and 25% (w/v) dilutions in sterile purified water and 50 and 25% (w/v) dilutions in catalase solution by agar well diffusion assay against MRSA

Code no.	Honey samples	Zone of inhibition (mm)			
		50% in water	50% in catalase	25% in water	25% in catalase
Standard	Manuka	16.0 ± 0.1	14.9 ± 0.0	12.0 ± 0.1	11.1 ± 0.1
H01	Sidr	17.2 ± 0.2	NZD^a^	10.2 ± 0.2	NZD
H02	Talha	13.4 ± 0.1	NZD	10.7 ± 0.2	NZD
H03	Sumra	18.1 ± 0.1	11.4 ± 0.4	13.0 ± 0.0	NZD
H04	Sidr	17.2 ± 0.4	NZD	10.9 ± 0.0	NZD
H05	Talha	14.5 ± 0.3	11.8 ± 0.3	11.1 ± 0.0	NZD
H06	Sumra	14.5 ± 0.3	NZD	10.0 ± 0.0	NZD
H07	Zahoor	11.8 ± 0.4	NZD	NZD	NZD
H08	Zahoor	17.5 ± 0.3	NZD	11.1 ± 0.1	NZD
H09	Zahoor	12.8 ± 0.3	NZD	NZD	NZD
H11	Sidr	12.6 ± 0.2	NZD	NZD	NZD

^a^NZD; no zone detected

The positive control, a vancomycin disc (30 μg), produced a 20.5 ± 0.9 mm inhibition zone against MRSA and a 21.8 ± 0.2 mm inhibition zone against MSSA, whereas the negative controls, catalase solution and sterile distilled water, did not produce any inhibition zone.

Sumra honey inhibited the growth of clinical isolates at 12 ± 0.0 dilution (v/v%), and Manuka honey did so at 14 ± 0.0 dilution (v/v%), in the microbroth dilution assay (Table 4). This result means that Sumra honey has more total or synergistic antibacterial activity than Manuka honey. A statistically significant difference (Kruskal-Wallis test, *p* = 0.00) was noted among the mean MICs of tested honeys against MRSA and ATCC reference strains. Moreover, there was also a statistically significant difference (Bonferroni post hoc test, *p* = 0.00) between the mean MICs of Sumra honey and Manuka honey assayed against the tested pathogens (Table 5).

**Table 4:** Minimum inhibitory concentrations (MICs) (%v/v) of honeys against clinical isolates of MRSA and ATCC standard strains

Code no.	Bacteria	Manuka	Sumra	Simulated
977414	MRSA-sputum	14 ± 0.0*	12 ± 0.0	35 ± 0.0
948815	MRSA-pleural fluid	14.6 ± 0.5	12 ± 0.0	35 ± 0.0
930401	MRSA-blood	14 ± 0.0	12 ± 0.0	35 ± 0.0
963905	MRSA-blood	14 ± 0.0	12 ± 0.0	35 ± 0.0
897648	MRSA-blood	14 ± 0.0	12 ± 0.0	35 ± 0.0
574577	MRSA-eye swab	14 ± 0.0	12 ± 0.0	35 ± 0.0
979101	MRSA-wound swab	14 ± 0.0	12 ± 0.0	35 ± 0.0
979638	MRSA-skin swab	14 ± 0.0	12 ± 0.0	35 ± 0.0
495310	MRSA-bronchial washing	14 ± 0.0	12 ± 0.0	35 ± 0.0
633489	MRSA- wound swab	14 ± 0.0	12 ± 0.0	35 ± 0.0
ATCC 43300	MRSA	14 ± 0.0	12 ± 0.0	35 ± 0.0
ATCC 29213	MSSA	14 ± 0.0	12 ± 0.0	35 ± 0.0
ATCC 25922	*Escherichia coli*	15 ± 0.0	15 ± 0.0	30 ± 0.0

*Mean of triplicate, with standard deviation

**Table 5:** Comparison of mean minimum inhibitory concentrations of Manuka, Sumra and simulated honey against MRSA

(I) Honey	(J) Honey	Mean Difference (I-J)	Std. Error	Sig.	95% Confidence interval	
					Lower Bound	Upper Bound
Manuka honey	Sumra honey	1.8^a^	.37	.000	.95	2.8
	Simulated honey	−20.4^a^	.37	.000	−21.4	−19.5
Sumra honey	Manuka honey	−1.8^a^	.37	.000	−2.8	−.95
	Simulated honey	−22.3^a^	.37	.000	−23.3	−21.4
Simulated honey	Manuka honey	20.4^a^	.37	.000	19.5	21.4
	Sumra honey	22.3^a^	.37	.000	21.4	23.3

^a^The mean difference is significant at the 0.05 level

## Discussion

Because of the increasing problem of antimicrobial resistance and on the basis of recent studies, honey is being integrated into modern medicine. There are several studies that reflect that a variety of beneficial effects of honey in wound healing originating from multiple bioactive compounds [42]. These effects encompass a wide range of benefits that are broad spectrum in nature, including avoidance of bacterial resistance, promotion of debridement and reduction in inflammation and malodour [9]. It is important to identify new honey with high therapeutic value for wound infections because a limited range of honey impregnated dressings are presently available, and they are quite expensive and not easily available everywhere [43]. Therefore, more research is required to identify new sources of honey from different countries so that patients can be benefitted with inexpensive, easily accessible and locally produced products.

In Saudi Arabia, there are more than 300 bee-associated floral species, including trees, shrubs, vines and herbs. However, Sumra (*Acacia tortilis*), Talha *(Acacia origena)*, Sidr (*Ziziphus spina-christi*), Dahiana (*Acacia asak*) and Lavendula species are the most important sources of honey production in the Taif, Al-Baha and Asir regions [44]. Both Acacia and Ziziphus species are drought and heat tolerant and are distributed in tropical and subtropical areas of Saudi Arabia [45]. Honey produced from Sidr trees is dark brown in colour and the is most popular and expensive because of its unique aroma and taste [46]. However, honey produced from Acacia species is consumed widely because of its medicinal and nutritive properties. Talha honey is light in colour (pale yellow), and Sumra honey is dark in colour (dark brown) [47].

Sumra (H03), Sidr (H04) and Zahoor (H08) honeys exhibited higher antibacterial activity in the agar well diffusion assay against MSSA at a 50% dilution in sterile distilled water than Manuka honey (Table 2). Four local honeys exhibited more antibacterial activity against MRSA than Manuka honey, including Sumra (H03), Sidr (H04), Sidr (H01) and Zahoor (H08) honeys (Table 3). This result means that these honeys have high H_2_O_2_ activity in comparison with that of Manuka honey. In some previous studies, Beri, Jarrah, Buckwheat and Ulmo honeys were identified as possessors of high H_2_O_2−_related antibacterial activity [26, 35, 48]. H_2_O_2_ is an important contributor to the antibacterial activity of honey and is present in variable concentrations in different honeys. However, the quantity of H_2_O_2_ present in honey is quite low (0.002 M) compared to that present in disinfectants (0.8 to 8 M) [49]. This small quantity of H_2_O_2_ in honey is unable to cause bacterial lysis when used in isolation. However, it has been shown that bacterial DNA degradation and eventual bacterial lysis requires an interaction between non-peroxide factors of honey (polyphenols and transition metals) and H_2_O_2_ [50, 51].

Apart from hydrogen peroxide, honey antimicrobial activity is also derived from other factors such as its acidity, high osmolarity and multiple non-peroxide plant-derived components [9, 52–55]. Recently, a number of bioactive compounds have been identified in honey with potent antibacterial properties. Important in this regard are methylglyoxal, leptosin, lysozyme, pinocembrin, 1,4-dihydroxybenzene and bee defensin-1 [52, 56–60]. The antibacterial activity of these substances is not destroyed by heat or catalase treatment, as hydrogen peroxide is. However, there is substantial variation in their concentration and presence in different honey samples [61].

The multiple antibacterial substances present in honey augment each other’s activities and produce synergistic effects on multiple targets of pathogenic bacteria [9]. This phenomenon is the probable reason that bacterial resistance to honey is difficult and not yet reported. In comparison, antibiotics usually consist of a single compound and have only one target in bacteria; therefore, it is easier for bacteria to generate resistance in stressful conditions [62]. It is important to identify bioactive non-peroxide components in honey so that new antibiotics could be designed and developed based on natural synergistic interactions of different components present in honey.

Sumra (H03) honey collected from Al-Baha, Beta Valley, and Talha (H05) honey collected from Al-Baha, Baljurashi, also exhibited an inhibition zone against MSSA at a 50% dilution in catalase solution in the agar well diffusion assay (Table 2). Since the catalase enzyme destroys hydrogen peroxide, both Sumra and Talha honeys possess non-peroxide antibacterial potential, similar to medically graded Manuka honey. However, their level of non-peroxide activity is low because they did not produce an inhibition zone at the 25% dilution in catalase solution, as did Manuka honey (Tables 2 and 3). It is important to detect active ingredients causing non-peroxide activity in Sumra and Talha honeys in future studies. Both Sumra (H03) and Talha (H05) honeys also showed non-peroxide activity against MRSA (Table 3). There are limited numbers of honey brands available worldwide with non-peroxide antibacterial activity, and the most researched honeys in this regard are Manuka and Medihoney. The non-peroxide activity of Manuka honey originates from a plant-derived compound known as methylglyoxal [63].

We also used a microbroth dilution assay for the determination of MICs. The dilution assay provides more precise and quantitative results than the agar well diffusion assay [64]. The agar well diffusion assay is widely used for evaluating the antibacterial activity of honey against bacterial pathogens; however, the assay has a number of limitations. These include a lack of sensitivity; large-sized plant-derived bioactive compounds present in honey may not diffuse at all or diffuse very slowly, thus being missed by this technique [65]. For instance, polymyxin, a well-known antibiotic that consists of a large-sized molecule poorly diffuses in the diffusion test; therefore, more sensitive assays, such as the broth dilution assay or agar dilution assay, are used for testing [66]. As the diffusion of honey is slow and the honey sample is further diluted by diffusion into the agar, bacteria can grow on the outer area before the inhibitory substance reaches them [67]. Moreover, non-polar substances may not readily diffuse through water-based agar [68]. A study revealed that there is a lack of a clear relationship between zone size obtained through agar diffusion assay and MIC evaluation in dilution methods [39]. These reports highlight that the agar diffusion assay may not be the most appropriate method for assessing the antibacterial activity of honey [39]. The results obtained through the agar diffusion assay are not truly representative of the overall antibacterial activity of any honey. Therefore, in this study, we also used a microbroth dilution assay to determine the MICs of Sumra (H03), Manuka and simulated honey against ten clinical isolates of MRSA and three reference strains.

Although the difference in the MICs of Manuka honey and Sumra honey was statistically significant, its clinical relevance and significance are not clear. Presently, a limited range of honeys has been approved by health regulatory authorities for the treatment of infected wounds and partial-thickness burns, which include Manuka and Medihoney. A number of in vitro studies have shown that there is less than a 5% difference in MICs of certified honeys and non-certified honeys [69, 70]. Moreover, Blair et al. (2009) revealed that both UMF honey (Manuka honey) and non-UMF honey are equally effective in overcoming bacterial resistance [71]. The clinical significance of this difference in MICs can be evaluated in randomized controlled clinical trials of registered honeys versus non-registered honeys for the treatment of infected wounds and burns in future studies.

Sumra honey had a lower MIC (indicative of better antibacterial activity) than Manuka honey against all tested clinical isolates and reference strains, MRSA (ATCC 43300) and MSSA (ATCC 29213). An equivalent MIC (15.0% v/v) was observed for *E. coli* (ATCC 25922) between Manuka honey and Sumra honey. No difference in MIC was found for MRSA and MSSA. This finding means that honey is equally effective against both methicillin-sensitive and -resistant types of isolates and has a unique mechanism of action against pathogenic bacteria. Interestingly, bacterial resistance to honey has been not documented, and this unique characteristic of honey makes it a valuable therapeutic agent for multidrug-resistant or pandrug-resistant pathogenic bacteria. This pattern was also observed in some previous studies [72, 73]. In comparison with previous studies, there is substantial variation among the MICs of Manuka honey for MRSA. For instance, a recently conducted study demonstrated that Manuka honey inhibited twenty-four MRSA isolates at 4.4% (v/v) [73]. Similarly, another study demonstrated that the growth of five MRSA strains was inhibited by Manuka at a 12.5% v/v dilution [48]. The variation in the MIC of Manuka honey against the same bacteria could be due to differences in the methodology used for MIC determination or the potency of the Manuka honey used. The MIC of simulated honey was recorded between 30 and 35% v/v (Table 4) against clinical isolates and ATCC reference strains. The results indicate that there are other factors besides sugar that contribute to the antibacterial activity of honey.

### Limitation of study

Physiochemical characteristics of tested honeys could not be determined due to the unavailability of the facility.

## Conclusions

Local honeys exhibited variable antibacterial activities against the tested pathogens. Sumra honey possesses better peroxide-based antibacterial activity than Manuka honey. Therefore, Sumra honey could be used as a potential therapeutic agent in those clinical conditions where additional peroxide activity is required. Moreover, it is important to conduct a large screening study in Saudi Arabia to identify honeys with high non-peroxide activity, such as Manuka honey.

## Data Availability

The data and materials used in this study are available upon request from the authors.

## Abbreviations

ATCC: American Type Culture Collection
MIC: Minimum inhibitory concentration
MRSA: Methicillin-resistant *Staphylococcus aureus*
MSSA: Methicillin-sensitive *Staphylococcus aureus*
UMF: Unique Manuka factor
